# Glycine receptor subunit-β-deficiency in a mouse model of spasticity results in attenuated physical performance, growth, and muscle strength

**DOI:** 10.1152/ajpregu.00242.2020

**Published:** 2022-02-02

**Authors:** Cintia Rivares, Alban Vignaud, Wendy Noort, Bastijn Koopmans, Maarten Loos, Mikhail Kalinichev, Richard T. Jaspers

**Affiliations:** ^1^Laboratory for Myology, Department of Human Movement Sciences, Faculty of Behavioral and Movement Sciences, Vrije Universiteit Amsterdam, Amsterdam Movement Sciences, Amsterdam, The Netherlands; ^2^Ipsen Innovation, Les Ulis, France; ^3^Sylics (Synaptologics BV), Amsterdam, The Netherlands

**Keywords:** physiological cross-sectional area, plantar flexor muscles, sarcomeres in series, skeletal muscle, spastic paresis

## Abstract

Spasticity is the most common neurological disorder associated with increased muscle contraction causing impaired movement and gait. The aim of this study was to characterize the physical performance, skeletal muscle function, and phenotype of mice with a hereditary spastic mutation (B6.Cg-Glrb^spa^/J). Motor function, gait, and physical activity of juvenile and adult spastic mice and the morphological, histological, and mechanical characteristics of their soleus and gastrocnemius medialis muscles were compared with those of their wild-type (WT) littermates. Spastic mice showed attenuated growth, impaired motor function, and low physical activity. Gait of spastic mice was characterized by a typical hopping pattern. Spastic mice showed lower muscle forces, which were related to the smaller physiological cross-sectional area of spastic muscles. The muscle-tendon complex length-force relationship of adult gastrocnemius medialis was shifted toward shorter lengths, which was explained by attenuated longitudinal tibia growth. Spastic gastrocnemius medialis was more fatigue resistant than WT gastrocnemius medialis. This was largely explained by a higher mitochondrial content in muscle fibers and relatively higher percentage of slow-type muscle fibers. Muscles of juvenile spastic mice showed similar differences compared with WT juvenile mice, but these were less pronounced than between adult mice. This study shows that in spastic mice, disturbed motor function and gait is likely to be the result of hyperactivity of skeletal muscle and impaired skeletal muscle growth, which progress with age.

## INTRODUCTION

Spastic paresis, due to an acquired perinatal upper motor neuron lesion (i.e., cerebral palsy) or genetic mutations (i.e., hereditary spastic paraplegia), is the most common cause of movement disorders in children (1). Cerebral palsy occurs in around 2 to 3 per 1,000 liveborn children, of whom 80% suffer from spasticity (2–4). Hereditary spastic paraplegia is less common than cerebral palsy, and its prevalence is still ∼3 to 10 per 10,000 liveborn children in Europe (5, 6). Children with spasticity due to cerebral palsy and children with hereditary spastic paraplegia show similar spasticity-related symptoms and secondary muscle adaptations [for review, see Fink (5)], which are treated similarly in both groups.

Children with spasticity experience overactivity of the muscle stretch reflex, which provokes resistance against joint extension (hyper-resistance) and is most prominent in the ankle plantar flexor and knee flexor muscles. Toe walking and crouch gait patterns are typical results of hyper-resistance (7–11). During locomotion, excessive contraction of spastic muscles contributes to the net torque over target joints. Consequently, antagonist muscles need to exert higher muscle force to execute particular movements, increasing their energy demand (12–15). Children with spasticity have reduced mobility and social participation (16–19); this ultimately results in lower daily physical activity compared with their typical developing peers (18, 19).

During development, spasticity-related hyper-resistance may affect skeletal muscle growth and adaptation, as well as that of connective tissue and bony structures (20–22). Several studies have shown impaired longitudinal fascicle growth in children with spasticity, particularly in lower limb muscles (23–26). The underlying mechanism for this may be an impaired longitudinal muscle fiber growth, as has been shown during development in children with spastic paresis (23, 27), which indicates that the number of sarcomere in series is likely lower in these fascicles. Therefore, spastic muscles are likely to operate at higher sarcomere lengths than those of typical developed muscles, which may explain the increased passive resistance (22, 28). Of note, since human lower extremities have a predominantly pennate morphology (29), longitudinal muscle growth also occurs through increases in the muscle fiber cross-sectional area (30–33). In addition to impaired muscle growth, structural alterations of collagen and the extracellular matrix may contribute to muscle hyper-resistance, as a result of their incrementing effect on the stiffness of muscle fiber bundles (28, 34). However, whether connective tissue content is increased in spastic muscle is subject to controversy and may differ between muscles (cf. Refs. 22, 28, and 35).

Clinical interventions aim to reduce muscle hyperactivity and increase joint range of motion. Interventions are temporally successful; however, studies show large variations in treatment outcomes (36, 37). Moreover, long-term functional limitations are recurrent (38). Improvement of interventions and development of new drugs require insight into mechanisms underlying spasticity. In vivo studies with typical developing children and children with spasticity provide limited detail of the underlying mechanistic causes. Animal models resembling the effects of spasticity in children are often used to test different treatments as they allow for more detailed insight into the multitude of determinants of hyper-resistance and impaired mobility. Findings of such studies are often extrapolated to the clinic. For example, Cosgrove and Graham (39) tested the effects of botulinum toxin injections to prevent hyper-resistance in calf muscles of spastic mice, the results of which were decisive for the current use of botulinum toxin injections in children with spasticity.

Although the etiology of impaired motor function of spastic mice is different from that of patients with neurological motor disorders, such as spastic cerebral palsy or hereditary spastic paraplegia, symptoms in spastic mice and patients with motor disorders are similar. Mice with glycine receptor unit β deficiency (also referred to as spastic mice) show reduced postsynaptic glycinergic neurotransmission, which affects the balance between excitation and inhibition of motor neurons. Reduced inhibition of motor neurons in spastic mice results in elevated spontaneous involuntary motor neuron activity, increased involuntary muscle contraction, malfunction of recurrent and reciprocal inhibition, as well as startles (40–46). These symptoms are similar to those of patients with spasticity. Spasticity being defined by the SPASM consortium as “a disordered sensori-motor control resulting from an upper motor neuron syndrome, presenting as intermittent or sustained involuntary activation of muscles” (47, 48). Such involuntary activation is associated with impaired motor control, muscle weakness, inappropriate reflex activity, loss of normal reciprocal inhibition, abnormal co-activation between agonist and antagonist muscles, as well as startle (49–56). Despite the etiological differences between spastic mice and patients with spasticity, this mouse model seems to represent the clinical features of what is generally referred to as spastic in neurological motor disorders. In spastic mice, the onset of these symptoms presents at an early age (2 wk of life) in mice that can reach an adult age (57, 58). This makes the spastic mouse model suitable for investigation of the mechanisms underlying spasticity-associated gait impairment, as well as the testing of novel interventions to improve physical performance (i.e., stem cells therapy, botulinum toxin injections, casting, and/or surgical interventions). However, the extent of behavioral changes and alteration in muscle function of this mouse model has not been quantified in detail.

The aim of the present study was threefold: *1*) to quantitatively characterize gait, motor function, and physical activity of spastic mice across postnatal development, at the age of 4, 6, 10, and 14 wk; *2*) to obtain insight into the mechanisms underlying spasticity-associated limitations in physical performance, in particular, contractile force characteristics and the morphological and histological phenotype of lower limb muscles (gastrocnemius medialis and soleus) in juveniles (6 wk) and adults (18 wk), as well as to assess the expression of growth factors in the gastrocnemius lateralis in adults; and *3*) to discuss our results in the context of human studies on the effects of spasticity to obtain insight into whether spasticity affects the growth, physical performance, and/or muscle function of humans and mice with spasticity in a similar way.

## MATERIALS AND METHODS

### Animals

Mice carrying a spontaneous mutation in the LINE-1 element insertion of the glycine receptor β-subunit (58) were obtained from a cryopreserved stock at Jackson laboratories (Stock 000066; B6.Cg-Glrb^spa^/J; Bar Harbour, ME). The colony was maintained on a C57Bl/6J background (Charles River Laboratories, L'Arbresle, France; European supplier of Jackson Laboratories) in the animal facilities of the Vrije Universiteit Amsterdam (Amsterdam, The Netherlands) and was backcrossed to C57Bl/6J at least every third generation. Experimental homozygous Glrb^spa^ mice and wild-type (WT) littermate controls were obtained by breeding heterozygous carriers. Food was provided on the cage bedding and long-spouted water bottles were used. At age 2 to 3 wk, ear tissue samples were taken for genotyping, and mice were weaned at age 3 to 4 wk. Before age 4 to 5 wk, ∼25% of homozygous mice had died prematurely or disappeared from the nests. Premature deaths were associated with extremely low body weight (∼5 g), whereas surviving homozygous mice had a considerably higher body weight (>7 g). A total of 15 spastic mice (8 females/7 males) and 15 WT mice (7 females/8 males) entered the behavioral test experiment at 4 wk of age; a subset of these mice were used for in situ experiments and tissue analyses (10 spastic/9 WT). Additional mice were bred for in situ experiments and tissue analyses at juvenile age (10 spastic/10 WT). Before weaning, mice were housed in individually ventilated cages and health status of the colony was confirmed to be specific pathogen-free (SPF) according to Federation of European Laboratory Animal Science Associations (FELASA) 2014 recommendations. After weaning, mice were moved to a barrier with conventional open cages under SPF conditions (following FELASA 2014 recommendations) with the exception of several pathogens not considered harmful for the immune competent C57BL/6J background used in this study (*Helicobacter typhlonius, Pasteurella pneumotropica, Chilomastix spp, Enta-moeba muris*, and *Murine Norovirus*). During both breeding and experiments, mice were maintained at 12-h light/dark cycle with lights on at 07:00 AM, humidity of 40%–60%, and room temperature of 19°C–22°C. Water and food were available ad libitum. Mice were group housed in Makrolon type 2 short cages on sawdust bedding with cardboard curls as nesting material, except during assessment of spontaneous behavior in the automated home-cage as described in *Spontaneous Behavior*. Behavioral experiments were performed during the light phase.

All experiments were approved by the Dutch Central Committee on Animal Experimentation (AVD112002016772) and in strict agreement with the guidelines and regulations concerning animal welfare recommended by Dutch law.

### Study Design

One cohort of 15 WT (male *n* = 8/female *n* = 7) and 15 spastic (male *n* = 8/female *n* = 7) mice were subjected to a series of behavioral tests at 4, 6, 10, and 14 wk. Body weight, neurological score, and righting reflex were scored every week. A subset of this first cohort of mice were used for in situ experiments and tissue analyses and included 9 WT (male *n* = 5/female *n* = 4) and 10 (male *n* = 4/female *n* = 6) spastic adult mice [aged (means ± SD) 17.9 ± 0.3 wk]. A second cohort of mice was used for in situ experiments and tissue analyses at juvenile age and included 12 WT (male *n* = 6/female *n* = 6) and 9 spastic (male *n* = 6/female *n* = 3) juveniles (aged 5.4 ± 0.2 wk).

### Spontaneous Behavior

Mice were individually housed in a PhenoTyper automated home-cages for 3 days as described earlier, with access to water, food, and a shelter, without human intervention (59). With respect to spontaneous behaviors in the first 3 days in the PhenoTyper, six groups of behavioral parameters were defined as described in *Spontaneous Activity in Home Cage* of the RESULTS and were analyzed using AHCODA (Sylics, Amsterdam, The Netherlands) with respect to temporal aspects, as previously described (59). Mice that built their nest outside the shelter (which interferes with calculation of all activity measures) were excluded. Mice that spent little time in the shelter (<60% of time in the shelter during light phase of *day 2* and *3*) in combination with being highly inactive outside the shelter (cumulative movement less than 2 cm/5 min for >25% of time outside during light phase of *day 2* and *3*) were classified as sleeping outside the shelter and excluded from the analyses.

### Neurological Score and Righting Reflex

General well being of the mice was assessed by observation of several characteristics. The neurological score was adapted from a scoring system originally developed to score the progression of a mouse model of amyotrophic lateral sclerosis (60). For the neurological score, three items were scored; extension reflex, curling toes, and tremor. With respect to extension reflex, a score of 0 was written down if a full extension of hind legs away from lateral midline was observed when mouse was suspended by its tail, and mouse could hold this for 2 s. If mice showed a partial extension reflex (*score 0.5*) or no extension reflex (*score 1*) this is taken as index of neurological symptoms. With respect to tremor, trembling of hind legs during tail suspension was scored (*score 0* = no tremor, *score 1* = tremor present). With respect to curling toes, the position of the toes was observed during forward motion (*score 0* = no curling toes, *score 1* = curling toes present). To calculate the neurological score, the average score on these three items was calculated. In addition, the original scoring system described a righting test (60). When a mouse is placed on its side and is unable to right itself within 30 s from either side this is considered a high neurological score. We noticed that spastic mice were unable to right themselves occasionally, and that this could last for more than 30 s, whereas on a next occasion the same mouse would right itself quickly. Hence, the righting test score was used a separate measure, and the actual righting time was reported as result.

### Grip Strength Measurements

Neuromuscular function was assessed by measuring the peak amount of force (N) mice applied in grasping a pull bar connected to a force transducer (1027DSM Grip Strength Meter, Columbus instruments, Columbus, OH). Mice were allowed to grasp the pull bar five times with front paws only, followed by grasping five times with front and hind paws.

### Motor Coordination

Motor function was evaluated using an accelerating rotarod (Roto-rod series 8, IITC Life Science, Woodland Hills, CA). In the first week of testing, mice received two habituation trials of 120 s (acceleration of from 0 to 20 rpm in 120 s) followed by three test trials (acceleration of from 0 to 40 rpm in 180 s) on the first day, and five test trials on the second day. During subsequent testing weeks, mice received one day of test trials only with five trials (acceleration of from 0 to 40 rpm in 180 s).

### Gait Analysis

Gait measurements were recorded and analyzed using the CatWalk system (Noldus IT, Wageningen, the Netherlands). Mice were introduced to the CatWalk system on *day 1* for habituation, and on the second day mice were tested. Several readouts were taken. Maximum contact area, i.e., maximum area of a paw that comes into contact with the glass plate, was recorded. Intensity (ranging from 0 to 255) of a print, i.e., the degree of contact between a paw and the glass plate (which increases with weight), was used to measure weight. Print length and width, i.e., the horizontal and vertical direction, respectively, of the complete print, were also recorded. The complete print is the sum of all contacts with the glass plate, as if the animal’s paw would have been inked, and print area is the surface area of the complete print. Stride length was defined as the distance between successive placements of the same paw. The regularity index, expressed as the number of normal step sequence patterns relative to the total number of paw placements, and base of support, i.e., the mean distance between either front paws or hind paws, were also recorded.

There were several exclusion criteria: noncompliant runs as defined by the CatWalk software (minimum run duration: 0.5 s, maximum run duration: 5.0 s; minimum number of compliant runs: 3 runs, minimum number of consecutive steps: 6; average speed between: 10 and 60 cm·s^−1^).

### Surgery and Preparation for in Situ Muscle Function Measurements

Mice received a subcutaneous injection of 0.1 mg/kg Temgesic (Buprenorphine, Reckitt Benckiser, UK) as an analgesic 20 min before surgery. Subsequently, they were anesthetized with 4% isoflurane, 0.2 L·min^−1^ O_2_, and 0.2 L·min^−1^ air. After nociceptive reflexes ceased, isoflurane levels were maintained at 0.5%–2.0%. Body temperature was maintained at 35°C using an electrical heating pad. To prevent the exposed muscles from dehydration, the lower leg was placed in a self-irrigating humidifier with a constant temperature of 35°C and a humidity of 80%–90%. On a regular basis, the exposed tissue was observed and, when needed, irrigated with isotonic saline.

Soleus and gastrocnemius medialis muscles were dissected free from surrounding tissue and from distal insertion while blood supply and innervation were maintained. Cuff electrodes were placed around gastrocnemius medialis and gastrocnemius lateralis branches of the tibial nerve, and served to stimulate the muscles. Finally, the femur was fixated at the distal condyle by a clamp.

### Experimental Setup and in Situ Muscle Force Measurements

To attain a full characterization of the calf muscles, we assessed for soleus and gastrocnemius medialis the following variables: *1*) length-force relationship, *2*) force-frequency relationship, *3*) fatigability, and *4*) rate of maximal force development. Contractions for all protocols were induced by supramaximal electrical stimulation of gastrocnemius medialis and gastrocnemius lateralis nerves for either soleus or gastrocnemius medialis muscle, respectively.

For assessment of the length-force relationship, tetanic contractions (150 Hz, 300 ms) were evoked at increasing muscle lengths (0.5 mm stepwise increase). Each tetanus was preceded by two twitches, to allow adjustment of muscle length. After each contraction muscles were allowed to rest for 2 min. Muscles were lengthened to ∼1 mm over their optimum length. Muscle/muscle-tendon complex lengths were calculated by measuring the distance between the markers placed on the proximal and distal parts of the muscle on the images made with a Panasonic HC-V720 camera. Two minutes after finishing the length-force protocol, muscles were stimulated by applying a 400-Hz, 150-ms pulse train to determine the rate of maximal force development.

The frequency-force protocol was started after a recovery period of 2 min at a short length. The muscle was stimulated at optimum length with increasing frequencies starting at 5 Hz and ending at 250 Hz each time during 300 ms. Finally, soleus and gastrocnemius medialis muscles were stimulated once every second for 2 min to test their fatigue resistance (soleus: 330 ms pulse train at 100 Hz, gastrocnemius medialis: 150 ms pulse train at 30–80 Hz, a frequency corresponding with 40% of optimum force). The mouse was euthanized by an overdose of 20% Euthasol 0.2 mL injected intracardially. Plantar flexor muscles together with tibialis anterior were carefully removed and aligned according to their muscle fiber arrangement (61). The right-sided soleus and gastrocnemius medialis muscles were covered with a thin layer of Tissue-Tek (Jung, Leica, Microsystems, Germany) and frozen in liquid nitrogen. The right gastrocnemius lateralis was frozen for gene-expression analysis. The left-sided plantar flexor muscles and tibialis anterior were fixed in formaldehyde and stored.

### Morphometric Analyses

The serial number of sarcomeres within muscle fibers was determined in left soleus and gastrocnemius medialis. Four proximal and four distal muscle fibers were isolated and mounted on glass slides with 50% glycerol, covered with a coverslip and sealed with nail polish as described by Heslinga and Huijing (31). Images were taken of every fiber using a bright-field microscope and a ×10 objective (Axioskop 50 microscope, Zeiss). Mean sarcomere length was determined by the number of sarcomeres over the entire muscle fiber length. The number of sarcomeres per fiber was calculated by dividing the measured muscle fiber length by the mean sarcomere length of the muscle fiber, using the method proposed by Tijs et al. (62). Optimum muscle fiber length was calculated using *Eq. 1*, in which optimum sarcomere length (2.2 μm) was taken as the mean value of the optimum sarcomere length in the length-force curve as described by Edman (63) and multiplied by the number of sarcomeres in series. Physiological cross-sectional area (PCSA) was calculated using *Eq. 2*, where muscle volume was obtained by *Eq. 3*. To obtain muscle volume, the muscle mass was multiplied by muscle density (ρ), which was set as 1.0597 g·cm^−3^, as suggested by Mendez and Keys (64).

(*1*) Optimum muscle fiber length [mm] = No. sarcomeres in series × 2.2 μm

(*2*) $$\text{PCSA }\left[{\text{mm}}^{\text{2}}\right]\;=\text{ muscle volume }\times {\text{ fiber length}}^{-\text{1}}$$

(*3*) $$\text{Muscle volume }\left[{\text{mm}}^{\text{3}}\right]\;=\text{ muscle mass }\times {\text{ ρ}}^{-\text{1}}$$

The tendon length at optimum muscle length was obtained from the images taken during length-force measurements by subtracting muscle belly length from gastrocnemius medialis muscle-tendon complex length at optimum length.

### Cryosectioning and Histology

Serial cross sections (10 μm thick) were cut from the mid-belly of soleus and gastrocnemius medialis using a cryostat (MICROM HM 550, GMI trusted laboratory solutions) at −20°C. Sections were mounted on glass slides (Menzel-Gläser, superfrost plus, Germany), air-dried, and stored at −80°C until further use. Three cross sections were stained for succinate dehydrogenase (SDH) after 15 min of air drying, as described previously (65). SDH activity was expressed as the absorbance at 660 nm·μm^−1^, section thickness per second of incubation time (Δ*A*660·μm^−1^·s^−1^).

### Immunohistochemical Staining of Myosin Heavy Chain Isoform Expression

Serial sections of soleus and gastrocnemius medialis were immunohistochemically stained against type I, IIA, IIB, and IIX myosin heavy chain (MHC) using primary monoclonal antibodies BAD5 (1 μg·mL^−1^), SC-71 (1 μg·mL^−1^), 6H1 (10 μg·mL^−1^), and BF-F3 (1 μg·mL^−1^) (Developmental Studies Hybridoma Bank), respectively. A double myosin fluorescence staining method was performed, as described by Bloemberg and Quadrilatero (66). After air drying for 10 min, sections were blocked with 10% normal goat serum for 60 min. Sections were incubated with a cocktail of two primary antibodies for 60 min. Subsequently, sections were washed in phosphate-buffered saline (PBS) for 5 min, which was repeated three times in total, and incubated in the dark with a cocktail of two secondary antibodies Alexa Fluor IgG2b 647/IgM 488, IgG1 488/IgM647 (Life Technologies, the Netherlands) for 60 min. After washing with PBS, sections were incubated with 2 μL wheat germ agglutinin (WGA, Life Technologies, the Netherlands) in 100 mL of PBS for 20 min, and washed again with PBS three times for 5 min each. Finally, sections were enclosed with Vectashield-hardset mounting medium with 4′,6-diamidino-2-phenylindole (DAPI; 1.5 μg·mL^−1^, Vector Laboratories). Images were captured at ×10 objective using a CCD camera (PCOl Sensicam, Kelheim, Germany) connected to a fluorescence microscope (Axiovert 200 M; Zeiss, Göttingen, Germany) with image processing software Slidebook 5.0 (Intelligent Image Innovations, Denver, CO). Muscle fiber type (MHC) was identified for as many fibers as possible. Hybrid fibers were taken into account (Fig. 4, *E* and *F*).

Muscle fiber cross-sectional area was manually measured using ImageJ (v. 1.4.3.67, National Institutes of Health, Bethesda, MD). The mean cross-sectional area of at least 50 fibers was determined. For soleus, muscle fiber cross-sectional area was determined per muscle fiber type, whereas for gastrocnemius medialis fiber cross-sectional area was determined in high and low oxidative regions without making a distinction between muscle fiber types.

### Quantification of Intramuscular Connective Tissue

Intramuscular connective tissue was determined and quantified as described previously (35). The sections were air-dried for 10 min and fixed with acetone for another 10 min at −20°C. Subsequently, the sections were fixated with bouin for 30 min and stained in Sirius Red saturated with picric acid for 30 min. After the sections were washed in 0.01 HCl, they were rinsed twice in ethanol absolute and twice in xylene (Bisolve Chimie, SARL, France). Thereafter, sections were enclosed with Entellan (Merck, The Netherlands). Binary images were taken of the stained cross section, so that red areas in the cross section were covered with black in the image.

The endomysium, connective tissue surrounding a single muscle fiber, was quantified by selecting ten regions containing only endomysium and muscle fibers. Mean endomysium thickness per muscle fiber (L_e_) was calculated by dividing the area endomysium per muscle fiber divided by the mean perimeter of the muscle fibers (for details see Ref. 35).

Three domains of perimysium were measured separately: length of collagen surrounding the smallest muscle fiber fascicles (*L*_p1_), length of larger fascicles that contain several primary fascicles (*L*_p2_), and length of collagen that traversed the cross section (*L*_p3_; traversing the cross section). Thicknesses were manually measured every 25 μm for at least five fascicles in *L*_p1_ and *L*_p2_, and 25 μm over a length of 1 mm. Since many of the cross sections were cleft, the thickness of the perimysium was not always measurable in each of the three domains.

### Myonuclear Density and Muscle Stem Cell Number per Muscle Fiber

To quantify myonuclear density and muscle stem cell number per muscle fiber, sections were co-stained with DAPI and anti-Pax7 antibodies according to van der Meer et al. (67). Sections were fixed in 4% formaldehyde in 1% PBS for 10 min, washed with PBS with Tween 20 (PBST) and blocked in 10% normal goat serum in PBS for 30 min. Subsequently, sections were incubated in 0.1% bovine serum albumin-PBS solution containing 4 μg·mL^−1^ Pax7 antibody (Developmental Studies Hybridoma Bank) in the dark for 60 min, washed in PBST, incubated 30 min with 4 μg·mL^−1^ Alexa Fluor 488 (1:500, Molecular Probes, Life Technologies) goat anti-mouse secondary antibody (Invitrogen, Breda, the Netherlands) and washed with PBST. Then sections were incubated in the dark for 20 min with 2 μL Texas red-WGA in 100 mL PBS conjugate (Invitrogen, Breda, the Netherlands) and washed with PBS. Finally, sections were enclosed with Vectashield-hardset mounting medium with DAPI (Vector Laboratories). Images were captured at ×10 objective using a CCD camera (PCOl Sensicam, Kelheim, Germany) connected to a fluorescence microscope (Axiovert 200 M; Zeiss, Göttingen, Germany) with image processing software Slidebook 5.0 (Intelligent Image Innovations, Denver, CO). The number of myonuclear fragments per muscle fiber cross section was manually counted in an average of 50 fibers per muscle, whereas muscle stem cell fragments per muscle fiber cross section were counted throughout the whole muscle cross section. To calculate the number of myonuclei (Mn) in muscle fiber, we used *Eq. 4*, as suggested by Jaspers et al. (68).

(*4*) $$M_{\text{n,f}}=\frac{N_{\text{n,s}}\times L_{\text{f}}}{L_{\text{m}}+D_{\text{s}}}$$

where *N*_n,s_ is the counted myonuclei per fiber cross section, *L*_f_ is the muscle fiber length, *L*_m_ is the length of the myonuclei, and *D*_s_ the thickness of the cross section. The length of myonuclei was assumed to be 12 μm (67). The myonuclear domain was obtained by dividing the fiber cross-sectional area by the myonuclei per 1 mm of fiber length. Number of muscle stem cells is reported as counted fractions per muscle fiber cross section of soleus and gastrocnemius medialis muscles.

### RNA Isolation and Real-Time PCR

RNA was extracted from frozen gastrocnemius lateralis muscles using TRIzol (Invitrogen) and homogenized with an automatic potter homogenizer (B. BRAUN, Melsungen, Germany) followed by RNA purification using the Ribo pure-kit (Thermo Fisher Scientific, Waltham, MA). RNA concentration and purity were measured using spectroscopy (Nanodrop 2000, Thermo Fisher Scientific, Wilmington, DE). RNA was reversed transcribed using SuperScript VILO MasterMix (Applied Biosystem, Carlsbad, CA) and the thermal cycler 2720 (Applied biosustems, CA). For each PCR target, 12.5 ng of the reverse transcription reaction product was amplified in duplicate using Fast SYBR Green master mix (Applied Biosystems). Real-time PCR was performed on a StepOne Real-Time PCR system (Applied Biosystems). For MuRF1, MAFbx, Mstn, IGF-1 Ea, MyoD, Pax7, and SDH mRNA, cycle threshold was subtracted from the mean cycle threshold value of 18S rRNA Δ*Ct* and converted into relative concentrations by 2^−Δ^*^Ct^*. The sequences for the primers (Invitrogen, the Netherlands) used for the specific RNA and mRNA targets are shown in Supplemental Table S1 (see https://doi.org/10.6084/m9.figshare.14838078).

### Data Treatment

MATLAB scripts were used for evaluation of the force data, and for calculation of number of sarcomeres in series (version R2017a, The MathWorks, Inc., MA). The public domain software ImageJ (version 1.52e, US National Institutes of Health, MD) was used to assess the quantity of endomysium, perimysium, number of myofiber, type, fiber cross-sectional area, and mean sarcomere lengths.

### Length-force characteristics.

Passive force was determined as the mean force between the second twitch and the tetanus and was expressed as a function of muscle length. This relation was fitted according to a second-order exponential (*Eq. 5*). Active muscle force was calculated by subtracting passive force at each muscle length from total muscle force. The active length-force was fitted by a polynomial function (*Eq. 6*)

(*5*) $$y=e^{b0\;+\;b\text{1}x}+\;b_{\text{2}}$$

(*6*) $$y=b_{0}+b_{\text{1}}x+b_{\text{2}}x^{\text{2}}+b_{\text{3}}x^{\text{3}}+b_{\text{4}}x^{\text{4}}+b_{n}x^{n}$$

In *Eqs. 5* and*6*, *b*_0_ to *b_n_* represent constants determined by the least square fitting procedure. To select the polynomial order that describes the length-force relationship most adequately, fits between a second and a fifth order were tested using one-way analysis of variance (ANOVA). The lowest polynomial order that yielded a significant improvement of the length-force fit was elected. Muscle optimum length and muscle active slack were defined as the muscle lengths at which the maximum of the polynomial was encountered, and the intercept of the fitted curve with the *x*-axis, respectively.

### Force-frequency relationship.

Calcium sensitivity in soleus and gastrocnemius medialis was assessed by electrical muscle stimulation over a range of frequencies (i.e., 5–250 Hz in adult and 10–125 Hz in juvenile mice) at the optimum length of the active muscle. Force-frequency relationship was obtained by subtracting the mean passive muscle force (measured between the second twitch and the tetanus) from the total force and dividing total force at each frequency by the maximal force.

### Excitation-contraction coupling.

Maximal rate of force development was described as the rate at which the active force went from zero to maximal active force and is expressed in Nm·ms^−1^.

### Endurance capacity.

Fatigability was assessed by calculating the decrease in active muscle force at the end of the series of contractions, expressed as the percentage of the maximal obtained force during the protocol.

### Statistics

Data were tested for normality and homogeneity and in case assumptions were not met, data were transformed [i.e., (natural)-log, exponential, or square-root transformation].

In juvenile mice, there was a significant difference in age between males and females, which was reflected in body and muscle masses. Therefore, we performed the statistics for all variables except force-frequency, sarcomeres in series, and histological parameters only on juvenile male mice.

A two-way mixed ANOVA was used to test for differences in physical performance, gait, length-force, and force-frequency data. In case effects of sex could not be tested, independent *t* tests between groups (juvenile mice) were performed. The effect of sex was excluded as explanatory factor from interaction effect when the number of pairwise comparisons for a variable exceeded the actual number of mice per group (e.g., for fiber types of gastrocnemius medialis muscle). Interaction effects and sex main effects were explicitly described when present, otherwise only group main effects were reported. General estimating equations were used to determine the relationship between righting time, neurological score, and righting reflex with age and age-sex interaction within the spastic mice group. These statistics did not include WT mice, as their score was constantly zero. Two-way ANOVA was used to test for differences in all the other parameters.

All statistical analyses were performed using SPSS (IBM SPSS statistics 25, NY). Prism (v. 8.2.0, GraphPad software Inc., CA) was used to plot the figures. Effects were considered significant at *P* < 0.05. Data are presented as the means ± standard deviation (SD). Behavioral data of this study will be published in the public repository of AHCODA-DB at https://public.sylics.com.

## RESULTS

### Body Weight and Tibial Length

Spastic mice had lower body weight compared with WT controls, both as adults and as juveniles. Moreover, female mice had lower body weight compared with male mice (Table 1). The tibia length of spastic mice was shorter than that of WT controls in both adult and juvenile animals. Adult female mice had shorter tibia length than adult male mice (Table 1).

**Table 1.** Body measurements of the adult and juvenile mice

	Adult WT		Adult Spastic		WT-Spastic	*P*	♂-♀	*P*
	♂*n* = 5	♀*n* = 3	♂*n* = 3	♀*n* = 5	Difference, %		Difference, %	
Age, wk	18.0 (1.7)	17.5 (1.7)	17.9 (0.9)	18.0 (1.3)		>0.05		>0.05
Body weight, g	32.8 (4.9)	22.3 (1.2)	24.0 (2.1)	19.8 (0.8)^a,b^	25	<0.05	17–31	<0.05
Tibia length, mm	18.89 (0.5)	18.5 (0.1)	18.2 (0.6)	17.7 (0.3)^a,b^	4.5	<0.01	3	<0.05

	Juvenile WT		Juvenile Spastic		WT-Spastic	*P*	♂-♀	*P*
	♂*n* = 6	♀*n* = 6	♂*n* = 6	♀*n* = 3	Difference, %		Difference, %	
Age, wk	6.4 (0.4)	4.6 (1.1)^b^	5.9 (0.7)	4.7 (0.6)^b^		>0.05		>0.05
Body weight, g	20.0 (1.1)	16.5 (1.1)^b^	15.8 (4.4)	12.0 (2.7)^a,b^	20	<0.05	16	<0.05
Tibia length, mm	17.3 (0.4)	16.1 (0.4)	15.5 (1.5)	14.7 (0.8)^a^	9	<0.05	9	<0.05

Differences between wild-type (WT) and spastic and between male and female (♂-♀) mice are shown in percentage, as well as *P* values belonging to these differences. Data are presented as means (SD).

^a^Differences between groups; ^b^within-groups, i.e. sex effect.

### Physical Performance

**Figure 1.**
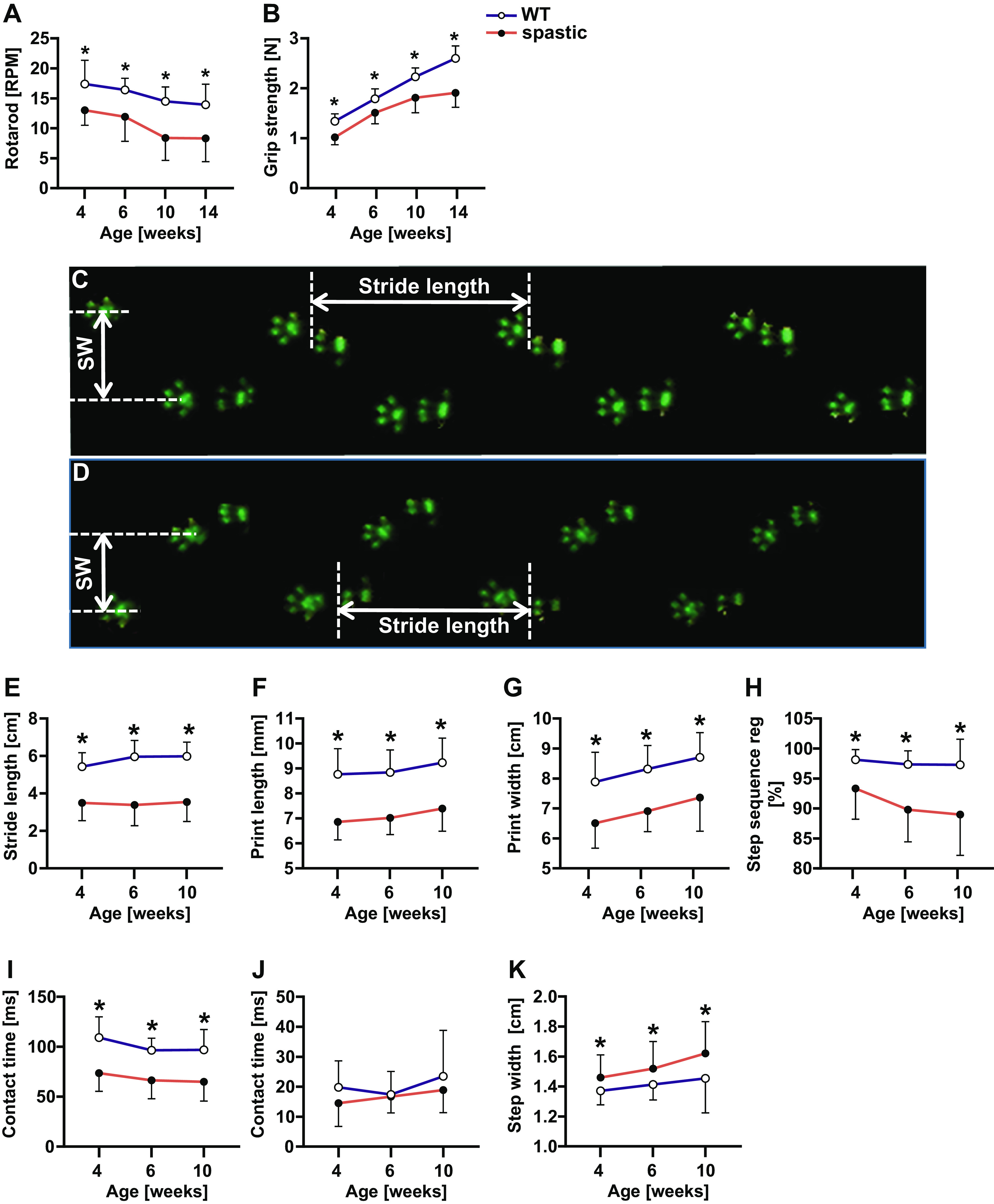
Motor function of spastic mice. *A*: rotarod performance. *B*: grip strength of front and hind limbs together. Typical footprints of wild-type (WT) mice as registered by the Catwalk (*C*) and typical examples of footprint of spastic mice as registered by the Catwalk (*D*), stride length (*E*), print length of hind limb (*F*), print width of hind limbs (*G*), step sequence regularity index (*H*), contact time of single limb (*I*), contact time of both hind limbs together (*J*), and step width of front limbs (*K*). Results are reported as means (SD). *Significant differences between WT and spastic mice.

Spastic mice fell from the rotarod at lower speed than WT controls, despite the reduction of rotarod performance over time in both genotypes (*P* < 0.0001; Fig. 1*A*). Spastic mice had lower grip force compared with WT controls, despite its increase over time (*P* < 0.0001) in both groups (Fig. 1*B*).

At all ages, spastic mice had a higher neurological score in comparison with WT controls (i.e., score of WT mice was zero), even though it was constant during growth. Spastic mice showed an attenuated righting reflex, whereas WT mice maintained their righting time below 1 s (*P* < 0.05). During growth, the righting time of spastic male mice increased by 0.78 s/wk (*P* < 0.05) and that of spastic female mice increased by 0.94 s/wk (*P* < 0.05).

### Gait

Gait of spastic mice was characterized by shorter stride lengths and a smaller print length and width compared with those of WT controls across all age groups (Fig. 1, *E–G*). Spastic mice showed an irregular order of front and hindlimb placements and shorter ground contact times of single limbs, but normal ground contact times of both limbs together when compared with WT controls (Fig. 1, *H*–*J*). The step width of the front paws increased with age in both spastic and WT mice, albeit with a lower rate in the former group (Fig. 1*K*).

### Spontaneous Activity in Home Cage

Spastic mice were less active during the dark phase and spent more time in the shelter compared with WT controls across all age groups (Table 2). The time spent in the shelter decreased over time in WT controls (*P* < 0.0001), while remaining stable in spastic mice. Also, spastic mice showed reduced physical activity, as a trend, during the light phase (Table 2). In addition, spastic mice showed reduced displacement velocity during activity bouts compared with WT controls (Table 2).

**Table 2.** Variables of body weight, behavior during growth, gait, and physical performance during growth

	4 Wk		6 Wk		10 Wk		14 Wk		
Variables	WT	spastic	WT	Spastic	WT	Spastic	WT	Spastic	*P*
Body weight, g	12.7 (1.9)	8.5 (4.5)*	19.4 (2.2)	15.5 (2.6)*	25.2 (3.4)	20.7 (3.4)*	26.4 (2.2)	21.1 (2.6)*	<0.001
Spontaneous activity									
Activity light, h	0.21 (1.50)	0.18 (0.20)	1.11 (0.67)	0.64 (0.23)	0.56 (0.56)	0.26 (0.17)	0.30 (0.41)	0.17 (0.10)	>0.05
Activity dark, h	2.02 (0.71)	1.19 (0.56)*	2.08 (0.56)	0.92 (0.30)*	3.22 (0.97)	1.70 (0.50)*	3.47 (1.16)	1.83 (0.44)*	<0.05
Sheltering dark, h	4.71 (1.38)	6.04 (1.76)	5.42 (1.27)	7.98 (1.76)*	3.61 (1.53)	5.74 (1.99)*	3.31 (1.01)	6.98 (2.46)*	<0.01
Displacement velocity, cm·s^−1^	22.72 (1.44)	18.82 (2.18)*	20.24 (1.84)	18.64 (1.74)*	20.95 (2.04)	14.90 (2.91)*	21.49 (1.98)	14.20 (2.78)*	<0.001

The table shows *P* value corresponding to group differences (main effect), whereas interaction effects or sex effects or changes over time are described in the text. Data are presented as means (SD).

* Post hoc differences.

### Contractile Force Characteristics of Soleus and Gastrocnemius Medialis Muscles

The aforementioned results show that spastic mice have abnormal gait, impaired motor function, and low physical activity. To obtain insight in the mechanisms underlying these characteristics, contractile force characteristics of plantar flexor muscles were analyzed.

#### Excitation-contraction coupling.

Spastic and WT adult mice had similar maximal rates of force development in the soleus and gastrocnemius medialis (Table 3). In female spastic mice, the absolute maximal rates of force development of gastrocnemius medialis were lower than in spastic males, and or WT females (Table 3).

**Table 3.** The maximal rate of force development in spastic and WT mice in two age groups

	Adult WT		Adult Spastic		Juvenile WT	Juvenile Spastic
	♂ *n* = 5	♀ *n* = 3	♂ *n* = 4	♀ *n* = 6	♂ *n* = 6	♂ *n* = 6
Soleus						
MRFD, mN·ms^−1^	1.62 (0.22)	1.37 (0.19)	1.47 (0.63)	1.05 (0.51)	1.05 (0.57)	1.01 (0.81)#
MRFD/*F*_max_, mN·ms^−1^·mN^−1^	6.74 (0.59)	6.75 (0.25)	9.58 (3.85)	7.61 (2.59)	7.92 (2.43)	14.05 (7.74)#
Gastrocnemius medialis						
MRFD, mN·ms^−1^	24.67 (4.64)	20.42 (6.98)	16.74 (9.70)	9.73 (4.53)^b^	22.56 (7.65)	10.23 (5.94)*
MRFD/*F*_max_, mN·ms^−1^·mN^−1^	20.47 (2.74)	18.36 (3.87)	18.39 (1.40)	14.74 (1.29)	27.33 (5.78)	16.43 (3.62)*

MRFD, maximal rate of force development. Data are presented as means (SD).

b Within-groups differences (i.e., sex effect), for adult mice, differences between groups were absent (♂-♀).

* Differences between WT and spastic mice in juvenile mice; #exclusion or absence of one animal.

Spastic and WT juvenile male mice had similar maximal rates of force development in the soleus muscle. In male spastic mice, absolute and normalized maximal rates of force development of gastrocnemius medialis were lower than in WT males (Table 3).

#### Force-frequency relationship.

Spastic and WT mice had similar active soleus and gastrocnemius medialis muscle forces as function of stimulation frequency at both juvenile and adult age (Supplemental Fig. S1; see https://doi.org/10.6084/m9.figshare.17695577).

#### Length-force characteristics of soleus and gastrocnemius medialis muscles.

**Figure 2.**
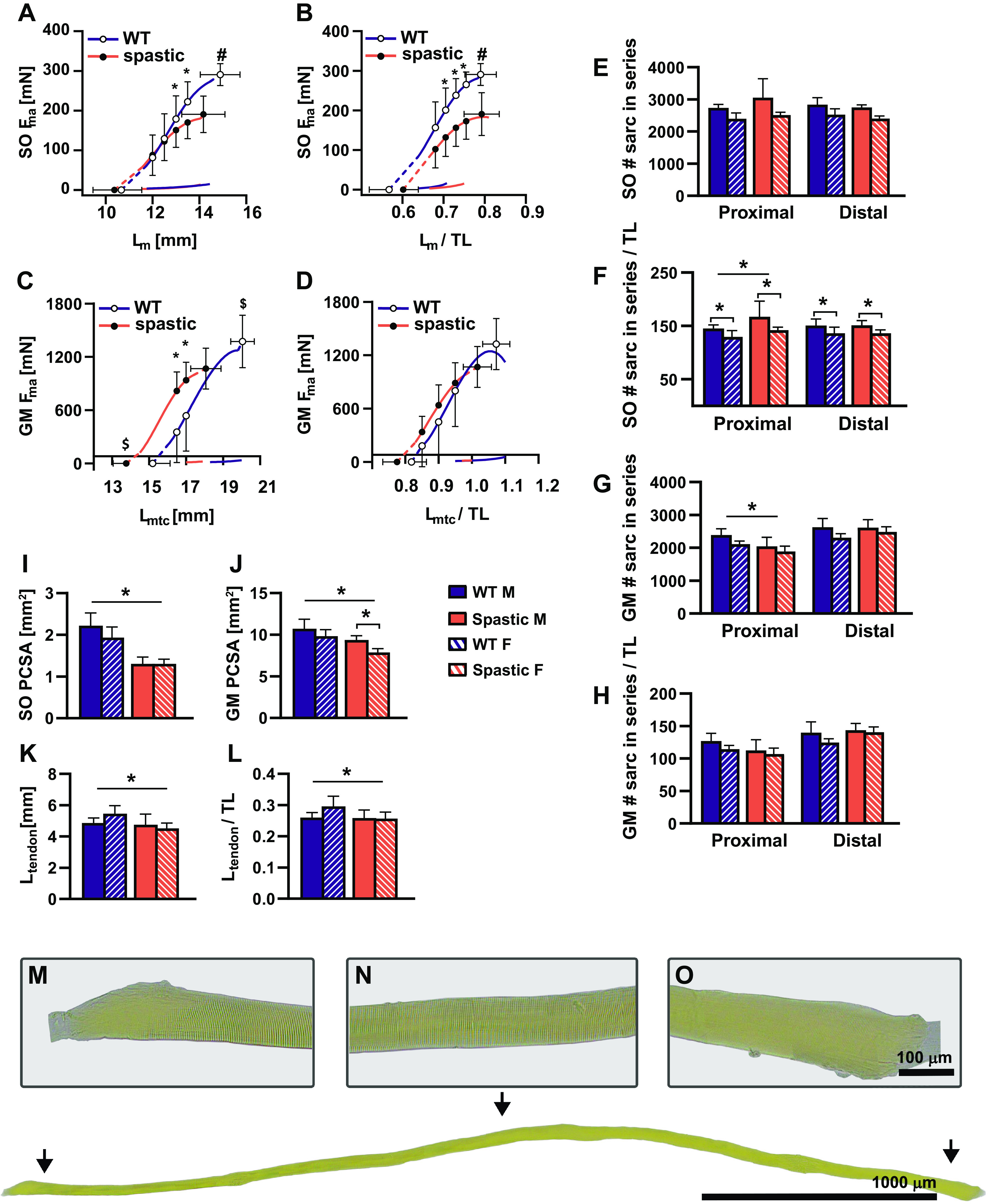
Effects of spasticity on adult muscle active and passive length-force relationship as well as on morphological determinants of muscle length and muscle force. Soleus (SO) absolute length-force relationship (*A*), SO length-force normalized for tibia length (TL) (*B*), gastrocnemius medialis (GM) absolute length-force relationship (*C*), GM length-force relationship normalized for tibia length (*D*), SO number of sarcomeres in series of proximal (Prox) and distal (Dist) fibers (*E*), SO number of sarcomeres in series of Prox and Dist fibers per unit of tibia length (*F*), GM number of sarcomeres in series of Prox and distal Dist fibers (*G*), GM number of sarcomeres in series of Prox and Dist fibers per unit of tibia length (*H*), SO physiological cross-sectional area (PCSA) (*I*), GM PCSA (*J*), Achilles tendon length (*K*), Achilles tendon length normalized for tibia length (*L*). *M*–*O*: an example of a muscle fiber with visible serial sarcomeres, the arrangement of actin and myosin filaments are clearly visible as light (I-band) and dark (A-band) cross striation. At the beginning and end of the fiber, the finger-like invaginations are shown. Results are reported as means (SD). *P < 0.05, #significant force at optimal muscle length differences between wild-type (WT) and spastic mice, and $significant slack and optimum muscle length differences between WT and spastic mice.

Adult spastic mice showed a 13% reduction in force at the ascending limb of the soleus length-force curve compared with WT controls (*P* < 0.05). Spastic mice also showed a 34% reduction in force of the soleus at optimum length compared with WT controls (Fig. 2*A*). Spastic and WT mice had similar muscle lengths at which force was actively exerted (Fig. 2, *A* and *B*).

Spastic mice had a 49% higher ascending limb of the gastrocnemius medialis length-force curve compared with WT controls, whereas force of the active muscle at optimum length was 22% lower than in WT controls (*P* < 0.05). Spastic mice had 10% shorter gastrocnemius medialis active slack and optimum lengths than WT controls, indicating that for gastrocnemius medialis the length-force curve was shifted toward shorter lengths (*P* < 0.05; Fig. 2*C*). Spastic and WT mice had similar muscle-tendon complex length when it was normalized to tibia length, which also yielded a shift of the spastic gastrocnemius medialis length-force curve toward the curve of WT gastrocnemius medialis, resulting in similar absolute forces (Fig. 1*D*). In addition, spastic and WT muscles had similar normalized active slack lengths and optimum length range of active force exertion (Table 4).

**Table 4.** Length-force relationships variables of Soleus and Gastrocnemius medialis muscles of spastic and WT mice of two age groups

	Adult WT		Adult Spastic		Juvenile WT	Juvenile Spastic
	♂ *n* = 5	♀ *n* = 3	♂ *n* = 4	♀ *n* = 6	♂ *n* = 6	♂ *n* = 6
Absolute length-force relationship values of Soleus muscle						
*F*_mao_, mN	294 (18)	285 (44)	219 (48)^a^	172 (37)^a^	199 (43)	119 (43)*
*L*_mao_, mm	15.2 (0.8)	14.4 (0.9)	14.5 (0.8)	13.9 (1.0)	15.5 (1.1)	13.3 (0.3)*
*L*_mas_, mm	11.1 (0.7)	9.9 (0.6)	10.6 (0.9)	10.2 (1.0)	10.5 (0.7)	8.9 (0.9)*
*L*_mas_ – *L*_mao_, mm	4.1 (0.7)	4.5 (1.3)	3.9 (0.6)	3.7 (0.6)	4.9 (0.7)	4.3 (0.7)
Normalized length-force relationship values of Soleus muscle						
F_mao_/BM, mN·g^−1^	9.1 (1.3)	12.7 (1.6)^b^	7.6 (2.6)	8.7 (1.9)^c^	10.1 (42.6)	7.4 (1.1)
*F*_mao_/PCSA, mN·mm^−2^	133.9 (11.9)	143.1 (25.4)	173.3 (57.7)	132.1 (28.0)	112.3 (23.3)	90.6 (33.3)
*L*_mao_/*L*_tibia_	0.80 (0.03)	0.77 (0.04)	0.80 (0.03)	0.79 (0.05)	0.91 (0.06)	0.87 (0.03)
*L*_mas_/*L*_tibia_	0.59 (0.04)	0.53 (0.03)	0.58 (0.03)	0.58 (0.06)	0.63 (0.03)	0.58 (0.03)*
*L*_mao_ – *L*_mas_/*L*_tibia_	0.21 (0.03)	0.24 (0.07)	0.22 (0.04)	0.21 (0.03)	0.28 (0.05)	0.29 (0.05)

	Adult WT		Adult Spastic		Juvenile WT	Juvenile Spastic
	♂ *n* = 5	♀ *n* = 3	♂ *n* = 3	♀ *n* = 5	♂ *n* = 6	♂ *n* = 6
Absolute length-force relationship values of the Gastrocnemius medialis muscle tendon complex						
*F*_mao_, mN	1,463 (234)	1,221 (380)^b^	1,282 (166)	939 (156)b	988 (284)	653 (341)
*L*_mao_, mm	19.9 (0.7)	20.4 (0.4)	18.7 (0.8)^a^^,^^b^	17.7 (0.6)^a^^,b^	18.1 (0.9)	16.0 (2.0)*
*L*_mas_, mm	14.6 (0.9)#	15.8 (0.3)	13.8 (0.4)^a^	13.6 (0.8)^a^	13.8 (1.3)	11.9 (1.6)*
*L*_mas_ – *L*_mao_, mm	5.2 (1.1)#	4.6 (0.2)	4.9 (0.7)	4.1 (0.6)	4.3 (0.5)	4.2 (1.1)
Normalized length-force relationship values of the Gastrocnemius medialis muscle tendon complex						
*F*_mao_/BM, mN·g^−1^	43.2 (5.5)	54.4 (14.8)	56.6 (4.5)	47.7 (9.6)	47.5 (10.7)	38.9 (14.8)
*F*_mao_/PCSA, mN·mm^−2^	136.4 (14.3)	124.1 (31.5)	137.5 (20.1)	117.9 (12.9)	12.9 (5.0)	10.1 (3.8)
*L*_mao_/*L*_tibia_	1.06 (0.04)	1.10 (0.00)	1.04 (0.02)	1.00 (0.05)^c^	1.05 (0.05)	1.03 (0.05)
*L*_mas_/*L*_tibia_	0.80 (0.04)#	0.85 (0.00)	0.77 (0.00)	0.77 (0.05)^c^	0.79 (0.06)	0.76 (0.04)
*L*_mas_ – *L*_mao_/*L*_tibia_	0.28 (0.06)#	0.25 (0.01)	0.26 (0.03)	0.23 (0.04)	0.26 (0.03)	0.27 (0.06)
Muscle volume						
SO, mm^3^	13.5 (1.4)	10.4 (1.1)^b^	8.3 (1.0)	7.0 (0.5)^a,b^	9.1 (1.1)	5.8 (1.5)*
GM, mm^3^	58.9 (5.1)	47.5 (5.2)^b^	47.8 (3.1)	39.5 (4.5)^a,b^	39.0 (5.7)	28.1 (9.7)*

GM, gastrocnemius medialis; *F*_mao_, force of the active muscle at optimum length; *L*_mao_, length of the active muscle at optimum length; *L*_mas_, length of the muscle in active slack; *L*_tibia_, tibia length; *L*_tendon_, tendon length; SO, soleus. Data are presented as means (SD).

a Differences between adult wild type (WT) and spastic mice muscles; ^b^differences between sex; ^c^interaction effect (i.e., group × sex).

# Exclusion or absence of one animal; *differences between juvenile WT and spastic mice muscle.

Juvenile spastic mice showed a 57% increase in the ascending limb of length-force curves of soleus and a 40% reduction of muscle force at optimum muscle length compared with corresponding WT controls (Table 4; *P* < 0.05). Spastic mice had a 15% lower soleus active slack length and a 14% lower optimum length compared with corresponding WT controls (*P* < 0.05). Normalization of muscle length by tibia length yielded similar curves for both groups, although for spastic soleus active slack length remained 8% shorter than for WT soleus (*P* < 0.05; Supplemental Fig. S2, *A* and *B*; see https://doi.org/10.6084/m9.figshare.17695619 and Table 4).

For juvenile spastic gastrocnemius medialis, length-force curves showed substantial variation in length range of active force exertion. Therefore, it was not possible to plot them together in one figure and calculate mean curves. Spastic and WT control mice had similar optimal gastrocnemius medialis muscle forces. In contrast, spastic mice showed a 14% and 12% shorter active slack length and optimum length, respectively, compared with WT controls (*P* < 0.05). Spastic and WT controls had similar active slack length and optimum length range, when these were normalized by tibia length (Supplemental Fig. S2*C*).

### Morphological Determinants of Muscle Length-Force Characteristics

The aforementioned results show that spastic plantar flexor muscles have lower muscle force and that the gastrocnemius medialis length-force curve was shifted toward shorter lengths. To obtain insight in the mechanisms underlying the attenuate muscle force, muscle morphology and histological phenotype were analyzed.

#### Muscle volume.

Adult spastic mice showed a 36% reduction in soleus volume compared with WT controls (*P* < 0.05; Table 4). Adult male mice had a 20% greater soleus volume compared with female mice (*P* < 0.001; Table 4). Similar results were observed for gastrocnemius medialis volume (i.e., 18% smaller in spastic mice than in WT mice). Note, that in both spastic and WT animals, females had an 18% smaller gastrocnemius medialis volume than males (*P* < 0.001; Table 4).

Juvenile spastic mice showed a 36% reduction in soleus muscle volume (*P* < 0.01) and a 28% reduction in gastrocnemius medialis volume (*P* < 0.05) than corresponding WT controls (Table 4).

#### Number of sarcomeres in series.

Spastic and WT adult mice had a similar number of sarcomeres in series in both proximal and distal regions of the soleus. After normalization for tibia length, spastic mice showed a higher number of sarcomeres in the proximal region of the soleus compared with the same region of WT soleus (*P* < 0.01; Fig. 2*E*). In addition, normalization yielded a difference between sex; WT and spastic female mice had fewer sarcomeres in series within the soleus compared with male WT and spastic mice (*P* < 0.05; Fig. 2*F*). For spastic male gastrocnemius medialis, the number of sarcomeres in series in the proximal region was slightly lower than in the WT (*P* < 0.05; Fig. 2*G*). After normalization for tibia length these differences were no longer apparent (Fig. 2*H*).

Spastic and WT juvenile mice had a similar number of sarcomeres in series in both proximal and distal regions of the soleus and gastrocnemius medialis (Supplemental Fig. S2, *E* and *F*).

#### Muscle physiological cross-sectional area.

Spastic adult mice had a 39% and 14% smaller soleus and gastrocnemius medialis physiological cross-sectional area, respectively, compared with WT adult mice (*P* < 0.05; Fig. 2, *I* and *J*).

Spastic juvenile male mice had a 26% smaller physiological cross-sectional area of soleus muscle compared with juvenile WT mice (*P* < 0.05), however, spastic and WT mice had similar physiological cross-sectional areas of gastrocnemius medialis (Supplemental Fig. S2, *D*–*G*).

#### Tendon length.

Spastic adult mice had shorter Achilles tendon length and shorter normalized Achilles tendon length (i.e., normalized for tibia length) compared with WT mice (Fig. 2, *K* and *L*).

Spastic juvenile mice had shorter Achilles tendon length compared with WT mice, however normalization of the tendon length yielded no differences between both groups (Supplemental Fig. S2, *J* and *K*).

#### Muscle fiber cross-sectional area.

**Figure 3.**
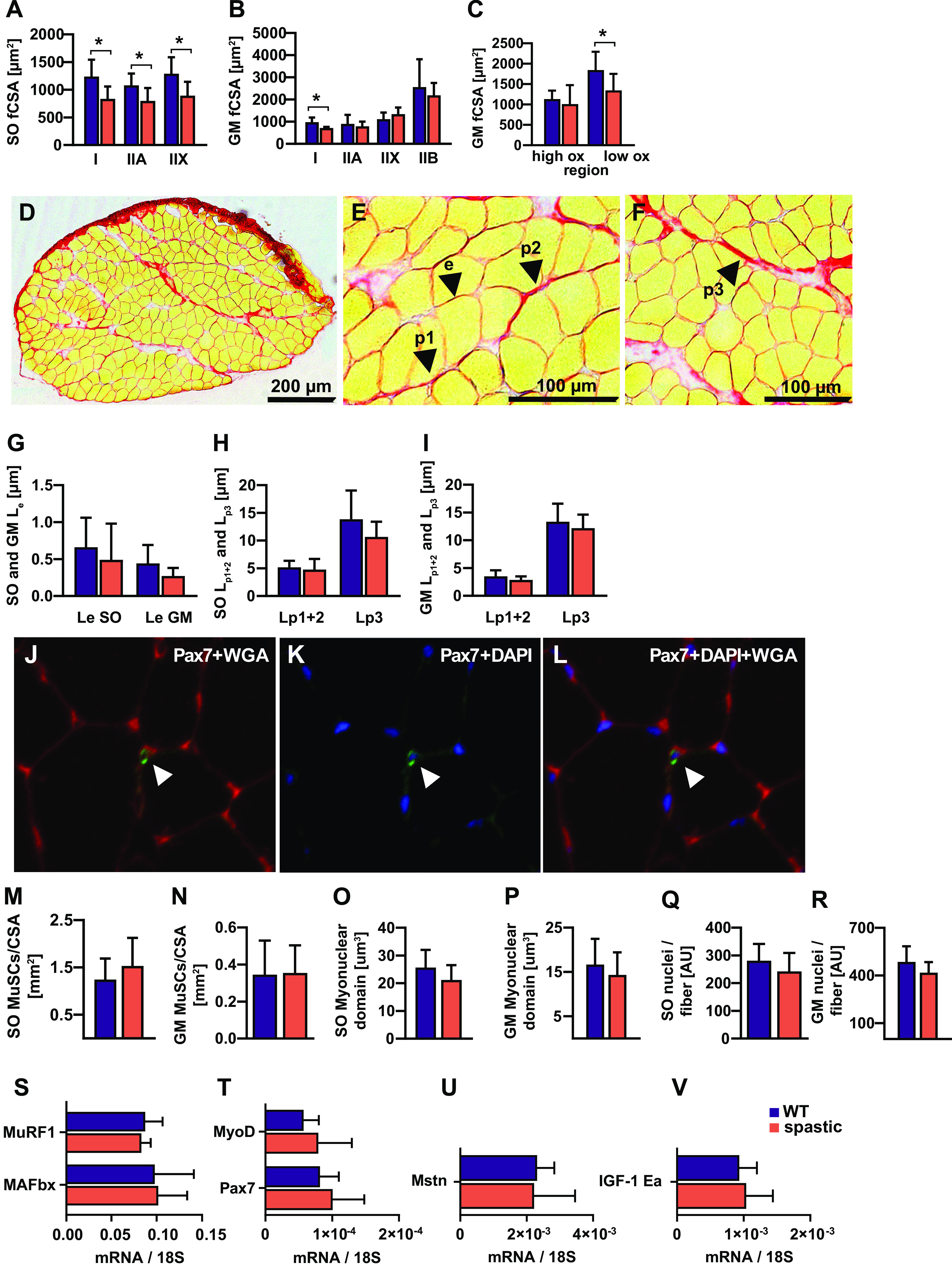
Active and passive muscle force determinants of adult spastic muscles. Soleus (SO) fiber cross-sectional area per fiber type (*A*), gastrocnemius medialis (GM) fiber cross-sectional area per fiber type (*B*), GM fiber cross-sectional area per muscle region (*C*), and typical example of a cross section stained with sirius red, connective tissue within a cross section is stained red, arrows show endomysium and perimysium (*D*–*F*). SO and GM endomysium length (*G*), SO perimysium length (*H*), GM perimysium length (*I*), representative images for stem cells identification with immunofluorescence staining (*J*–*L*). Labeled for pax7 (*J*; green), DAPI (*K*; blue), and Basal Lamina stained using wheat germ agglutinin (WGA) (*L*; red). SO muscle stem cells (MuSCs) per cross-sectional area (*M*), GM muscle stem cells per cross-sectional area (*N*), SO myonuclear domain (*O*), GM myonuclear domain (*P*), SO nuclei per fiber (*Q*), GM nuclei per fiber (*R*). *S* and *V*: gene expression levels of factors involved in the regulation of muscle fiber size relative to the expression of 18S RNA. Results are reported as means (SD). *Significant differences between wild-type (WT) and spastic mice. Ox: oxidative.

Spastic adult mice had, for all soleus muscle fiber types, smaller fiber cross-sectional areas compared with WT controls (Fig. 3*A*).

Spastic adult mice had, for gastrocnemius medialis type I muscle fibers, a smaller fiber cross-sectional area compared with WT controls (Fig. 3*B*). However, the cross-sectional areas of type IIA, IIX, and IIB were similar between both groups. In addition, spastic mice had a smaller cross-sectional area of fibers in the lox oxidative gastrocnemius medialis muscle region (consisting mainly of type IIB and IIX fibers) compared with WT controls (Fig. 3*C*). Moreover, the total cross-sectional area of fibers in the high oxidative muscle region was similar between both groups.

Spastic juvenile mice had a smaller fiber cross-sectional area of soleus type I fibers compared with WT juvenile controls (Supplemental Fig. S3*A*; see https://doi.org/10.6084/m9.figshare.17695622). In both the soleus and gastrocnemius medialis, a trend was observed toward a smaller fiber cross-sectional area in juvenile spastic fibers, although no significant differences were shown for fiber cross-sectional area of other muscle fiber types (Supplemental Fig. S3, *A*–*C*).

#### Intramuscular connective tissue content.

Spastic and WT mice had similar endomysium and perimysium thickness as well as fraction of endomysium per unit cross-sectional area and amount of endomysium per muscle fiber in both adult and juvenile muscles (Fig. 3, *G*–*I* and Supplemental Fig. S3, *D*–*F*).

#### Myotrophic determinants.

The aforementioned results show that spastic plantar flexor muscles are smaller in volumes and physiological cross-sectional area. To obtain insight in the mechanisms underlying the attenuated muscle growth, muscle stem cell, and myonuclear densities were analyzed as well as expression of myogenic regulatory factors, growth factors, and enzymes involved in degradation of muscle protein.

In both adult and juvenile mice, the number of Pax7-stained muscle stem cells per cross-sectional area in the soleus and gastrocnemius medialis was similar for WT and spastic muscles (Fig. 3, *M* and *N* and Supplemental Fig. S3, *G* and *J*).

In adult spastic soleus and gastrocnemius medialis, numbers of myonuclei per myofiber and the myonuclear domain were similar to those of WT mice (Fig. 3, *O*–*R*). Results from juvenile muscles show that the lack of difference between spastic and WT muscle is not age dependent (Supplemental Fig. S3, *H* and *I*, and *K* and *L*). The smaller physiological cross-sectional area in spastic mice could therefore not be explained by a reduced density of muscle stem cell myonuclei.

Figure 3, *S*–*V* shows for gastrocnemius lateralis mRNA levels of growth factors (IGF-1Ea and myostatin) and E3-ligases (i.e., Murf1 and MAFbx) involved in the degradation of muscle protein. Expression levels of these genes did not differ between spastic and WT muscle. These observations indicate that morphological differences between WT and spastic muscles are likely neither explained by elevated expression of genes involved in muscle protein degradation nor by a diminished growth factor expression.

### Determinants for Muscle Endurance

#### Muscle fatigue resistance and determinants of endurance capacity.

**Figure 4.**
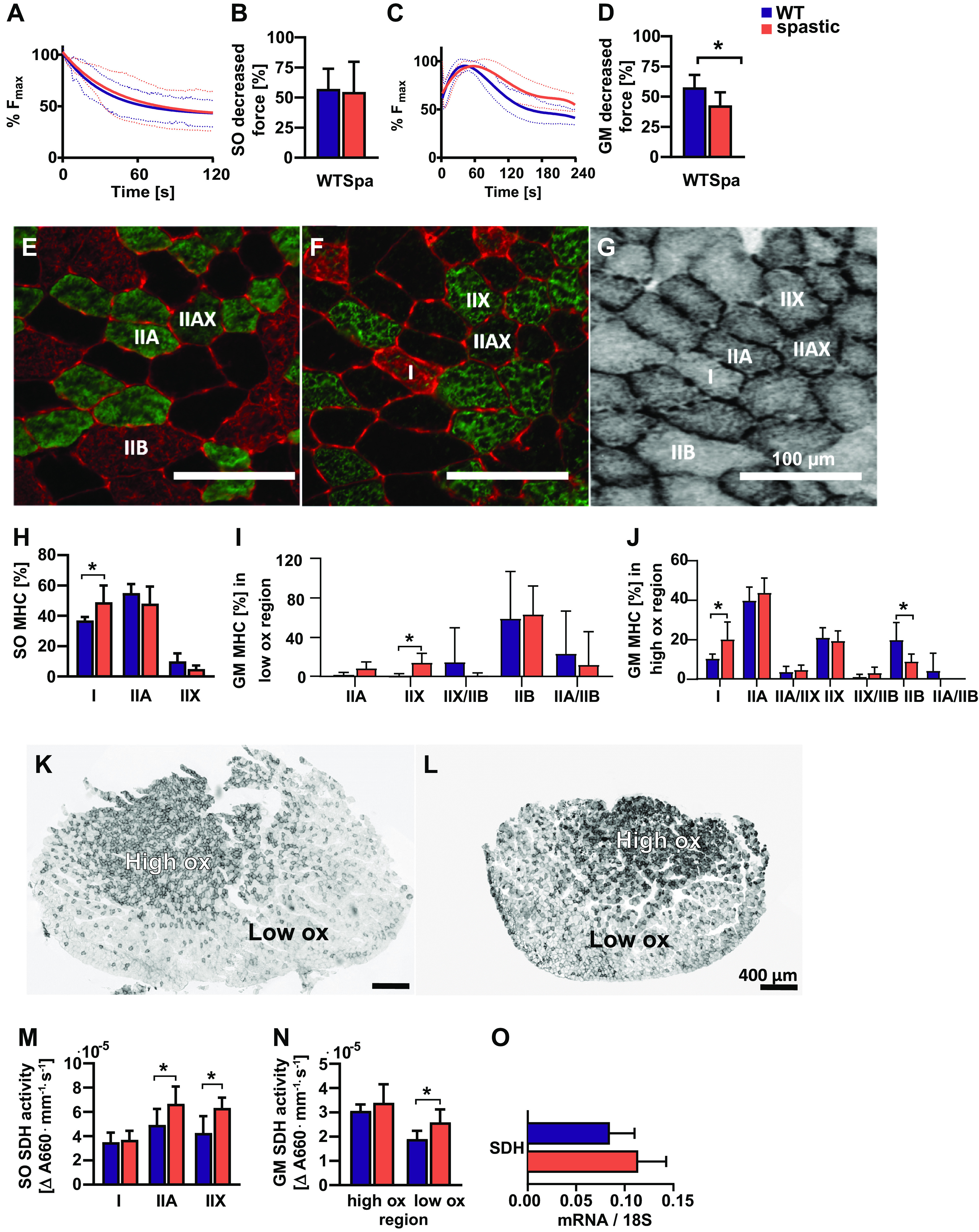
Endurance capacity of adult soleus (SO) and gastrocnemius medialis (GM) as well as endurance determinants. SO endurance curve (*A*), SO percentage of decreased force at the end of the fatigue protocol (*B*), GM endurance curve (*C*), GM percentage of decreased force at the end of the fatigue protocol (*D*), typical example of muscle fiber-type matching with SDH stained cross section (*E*–*G*). SO MHC distribution (*H*), GM myosin heavy chain (MHC) distribution in the low oxidative muscle region (*I*), GM MHC distribution in the high oxidative muscle region (*J*), typical examples of WT and spastic GM muscles, respectively (*K* and *L*). SO SDH activity (*M*), GM SDH activity (*N*), gene expression factors involved in the regulation of muscle metabolism, expression factors are relative to the expression of 18S RNA (*O*). Results are reported as means (SD). *Significant differences between WT and spastic mice.

In adult mice, the fatigue resistance of spastic and WT soleus was similar, as during 2 min of stimulation the percentage of decrease in force was similar in both groups (Fig. 4, *A* and *B*).

In adult spastic mice, the decrease in maximal force of gastrocnemius medialis after 4 min of stimulation was 16% lower than that of WT mice (*P* < 0.01; Fig. 4, *C* and *D*). These results show that spastic gastrocnemius medialis was more fatigue resistant than WT control.

Spastic and WT juvenile mice showed similar decreases in maximal force of soleus and gastrocnemius medialis. Although endurance curves for the soleus and gastrocnemius medialis showed later fatigue onset in spastic muscles, the total decrease in force did not differ between groups (Supplemental Fig. S4, *A*–*D*; see https://doi.org/10.6084/m9.figshare.17695625).

The aforementioned results show that spastic gastrocnemius medialis muscles were more fatigue resistant compared with WT muscles. To obtain insight in the mechanisms underlying the increased fatigue resistance, muscle fiber typing and mitochondrial density were analyzed.

#### Muscle fiber typing.

In adult spastic mice the percentage of type I myosin heavy chain (MHC) fibers of soleus was 27% higher than in WT controls (*P* < 0.01; Fig. 4*H*). The higher percentage of type I MHC in spastic soleus was mainly caused by a sex effect. Spastic adult female mice had a 30% higher percentage of type I MHC compared with spastic adult male mice, and a 34% higher than WT female (*P* < 0.001).

For gastrocnemius medialis muscle, the percentage of type IIX within the low oxidative region, of spastic mice was higher than that of WT gastrocnemius medialis (*P* < 0.01; Fig. 4*I*). Within the high oxidative region of spastic muscle, the percentage of type I fibers was 46% higher and type IIB fibers was 55% fewer than within this region of WT muscles (*P* < 0.05; Fig. 4*J*).

In juvenile soleus, muscle fiber typing showed similar distributions in both groups (Supplemental Fig. S4*E*). Within the high oxidative region of the spastic gastrocnemius medialis muscle, the percentage of type IIB fibers was 14% lower than within this region of WT juvenile muscles (*P* < 0.05; Supplemental Fig. S4, *F* and *G*).

#### Succinate dehydrogenase activity.

In adult spastic mice, the SDH-activity in type IIA and IIX muscle fibers of soleus was higher than in WT controls (*P* < 0.05; Fig. 4*M*).

In adult animals, spastic mice showed a 27% higher SDH-activity of muscle fibers within the low oxidative region of spastic gastrocnemius medialis than that of same type of fibers within WT gastrocnemius medialis (*P* < 0.01; Fig. 4*N*). SDH activity in the high oxidative muscle region of gastrocnemius medialis was similar in WT and spastic mice.

SDH activity in type I, IIA, and IIX fibers in spastic juvenile soleus was higher than that in similar fiber types in WT soleus (*P* < 0.05; Supplemental Fig. S4*H*). Gastrocnemius medialis SDH activity did not differ between juvenile WT and spastic mice (Supplemental Fig. S4*I*). Figure 4*O* shows mRNA levels for SDH expressed relative to 18S RNA levels for the gastrocnemius lateralis, which were similar in spastic and WT mice.

## DISCUSSION

The aim of this study was to quantify, in detail, the physical performance, physical activity, and gait of spastic (mice with a hereditary spastic mutation, which results in a reduced amount of glycine receptors β-subunit) compared with WT mice, as well as to characterize their soleus and gastrocnemius medialis calf muscles with respect to their contractile force characteristic, morphology, and histological phenotype at both juvenile and adult ages. We chose these two plantar flexor muscles because of their differences in geometry and function. In rodents, the soleus is a parallel-fibered muscle and its main function is to control posture, whereas the gastrocnemius medialis is a highly pennate muscle primarily involved in the push off during locomotion. Moreover, results of the present study are discussed and compared with those of human studies to obtain insight in whether spasticity affects the growth, physical performance, and/or muscle function of humans and mice with spasticity in a similar way.

### Effects of Spasticity on Growth

Here, we showed that growth of spastic mice is attenuated compared with WT mice (i.e., lower body mass at juvenile age and shorter tibia length at juvenile and adult age). Several factors might be responsible for the observed growth deficit (e.g., malnutrition, growth hormones deficiency, low level of physical activity, and intrinsic genetic factors). In spastic mice, muscle mRNA expression levels of factors associated with protein synthesis and degradation were similar to those of the controls. Therefore, it is not likely that the attenuated growth of spastic mice and their muscles is caused by altered regulation of protein synthesis, nor by increased degradation. The observed attenuated growth may be due to low levels of physical activity, as was seen for spastic mice in the home cage. However, the contribution of malnutrition and intrinsic factors cannot be excluded. Children with spasticity also show attenuated growth, which is manifested in a lower body weight and a shorter stature than their typical developing peers (69–72). In these children, attenuated growth is associated with malnutrition due to feeding difficulties and diminished serum levels of growth hormones (71, 73, 74). In addition, the total daily physical activity of children with spasticity is reduced compared with that of typical developing children (75). Children with spasticity and spastic mice may both experience reduced stimulus for growth due to low levels of physical activity.

### Effects of Spasticity on Physical Performance

Spastic mice showed impaired motor function, lower physical activity, and altered gait compared with WT mice. During walking, spastic mice showed a typical hopping pattern, characterized by shorter stride lengths, less and irregular surface-paw contact, as well as increased base of support area of front paws. These gait characteristics indicate that the coordination and balance control are hampered. In addition, shorter stride lengths in spastic mice are likely not the result of attenuated growth, as tibia length was 9% smaller in juvenile spastic mice compared with WT mice, and only 4.5% smaller in adult spastic mice (see Table 1). These differences do not explain the 36%–43% lower stride length in spastic mice at the age from 4 to 10 wk (see Fig. 1). Moreover, stride lengths in both growing WT and spastic mice did not increase as function of age, indicating that tibia length is likely not a key factor determining stride length. Low gait velocity and short step length are also characteristic of children with spasticity (13). In addition, these children show lower dynamic balance capacity compared with typical developing children (76). These latter characteristics combined with weak lower extremity muscles (77) imply a high energy demand during walking, which explains an early onset of fatigue (78). In children with spasticity, impaired mobility in combination with reduced exercise tolerance results in participation restrictions as well as activity limitations and consequently lower total daily physical activity (16, 19, 75, 79, 80). Although the exact reason for the observed low physical activity in mice is not clear, the impact of low physical activity on the muscle development of both spastic mice and children with spasticity appears similar.

### Effects of Spasticity on Muscle Length Range of Force Exertion

The length range over which a muscle is able to generate force is determined by several factors: the length of myofilaments, number of sarcomeres in series, number and length distribution of sarcomeres within muscle fibers, change in fiber and aponeurosis angle with change in muscle length, as well as the length and mechanical properties of tendinous structures within the muscle-tendon complex (81–84). In pennate muscles, an additional determinant to account for is the fibers’ cross-sectional area, as the length component of the physiological cross-sectional area co-determines muscle belly length (30, 32, 81, 85).

Spastic and WT mice showed a similar absolute and normalized length range of active force exertion of the soleus muscle. This lack of difference is in line with the observed lack of difference in the number of sarcomeres in series between spastic and WT soleus. Similar results were found in the soleus length-force characteristics of spastic rats (86). These results indicate that in rodent soleus muscle, the length range of active force exertion is not affected by spasticity. In children with spasticity it is unknown how the soleus muscle length differs from that of typical developing children.

The spastic gastrocnemius medialis muscle-tendon complex showed a shift in the absolute length range of active force exertion toward shorter lengths, whereas the length range of active force exertion was similar to that of WT controls. This shift was caused by a shorter Achilles tendon, a lower number of sarcomeres in series of the proximal muscle region and a smaller physiological cross-sectional area. Spastic and WT mice had similar gastrocnemius medialis normalized muscle-tendon complex lengths (by tibia length). This effect of normalization was in line with similar normalized serial sarcomere numbers observed between spastic and WT gastrocnemius medialis, which indicates that the attenuated longitudinal growth of the tibia in spastic mice results in reduced growth stimulus for the plantar flexor muscles and Achilles tendon.

In children with spasticity, growth of the spastic gastrocnemius muscle-tendon complex is different from that in typical developing children. The gastrocnemius medialis muscle fascicle and muscle belly lengths are lower and their Achilles tendon is in some studies reported to be longer compared with typical developing children (23–26, 87–89). The morphological and geometrical adaptations causing hyper-resistance of spastic muscles are highly heterogeneous, and depend on several factors, including: severity and location of the brain lesion, affected body part(s) (e.g., diplegic/quadriplegic), as well as growth and intervention history (20, 90–92). Moreover, morphological and geometrical adaptations within and between spastic muscles seem to differ among children with spasticity (cf. Refs. 89 and 93). Within the current spastic mouse model, morphological adaptations were homogenous, and muscle morphology was independent of the severity of motor dysfunction (i.e., neurological score and righting reflex) and intervention history. Therefore, we suggest that the muscle length range over which force is actively exerted is mainly affected by attenuated growth.

### Effects of Spasticity on Muscle Force Generating Capacity

In this study, we showed that the force generating capacity of spastic soleus is lower than that of WT mice. Moreover, a trend toward lower muscle force of the spastic gastrocnemius medialis muscle was observed. Lower force values are explained by the smaller physiological cross-sectional area of spastic muscles. Normalization of forces by physiological cross-sectional area (yielding specific muscle force) showed that reductions in force and physiological cross-sectional area were in the same order of magnitude. This indicates that spasticity caused a radial muscle fiber growth deficit, rather than an impaired force-generating capacity of the muscle (i.e., reduced quality). The present study also showed that in juvenile spastic mice, an increase in physiological cross-sectional area is less affected than in adult mice. Our results are in line with those of ultrasound studies performed on muscles from children with spasticity, as these children in general showed a reduced physiological cross-sectional area compared with typical developing children (27, 94, 95). Moreover, ultrasound studies have shown that at young age, the calf muscle morphology of children with spasticity is only slightly different from that of typical developing children (93). However from the age of 6 yr there is a slower increase of physiological cross-sectional area (87). Spastic mice and children with spasticity show similar attenuated increases in physiological cross-sectional area of spastic muscles.

### Effects of Spasticity on Passive Length Force Characteristics

Passive force as a function of muscle length is determined by slack length and stiffness of muscle belly and tendon (96), or more specifically by the individual components: number of sarcomeres in series, total number of sarcomeres arranged in parallel, tendon(s) length, and connective tissue content and its mechanical material properties (particularly of the perimysium).

Spastic muscles had smaller physiological cross-sectional areas and shorter Achilles tendon lengths, as well as similar numbers of sarcomeres in series and connective tissue content compared with WT controls; therefore, one would expect a reduced stiffness in spastic muscles. The lack of difference in muscle and tendon stiffness between spastic and WT muscles may be explained by spastic muscles having relatively more collagen type 1 (i.e., collagen type 1 is stiffer than type 3 collagen) and/or more cross linking between collagen fibrils. In addition, the lack of difference in passive properties between spastic and WT muscles suggests that the typical hopping gait pattern, with shorter stride lengths and less surface-paw contact, is mainly caused by the overactive stretch reflex rather than by enhanced passive stiffness due to secondary morphological adaptation of triceps surae muscles.

In children with spasticity, muscle-tendon complex stiffness is likely not caused by an increased number of parallel arranged sarcomeres as the physiological cross-sectional area of spastic muscles is generally smaller. However, it is possible that a reduced number of sarcomeres in series contribute to muscle stiffness. In some studies, Achilles tendon in children with spasticity has been reported to be longer than that in typical developing children (cf. Refs. 23–27 and 87–89). This indicates that in the spastic muscle-tendon complex, the tendon likely does not contribute to the increased muscle stiffness. In some muscles, the connective tissue content has been reported to be increased in some children with spasticity, however, large variations in connective tissue content between muscles and patients exist (e.g., see Refs. 28, 35, and 97). A possible explanation for this could be the severity of spasticity, as this seems to be related to the amount of connective tissue content (97).

### Effects of Spasticity on Muscle Endurance Capacity, Muscle Fiber Type, and Oxidative Metabolism

The gastrocnemius medialis of spastic mice was more fatigue resistant than that of WT controls. This could be largely explained by the higher mitochondrial content of spastic muscles and relatively larger percentage of slow type muscle fibers. The increase in mitochondrial density and muscle fiber type shift are in line with the observed smaller muscle fibers in spastic muscles, as the oxidative capacity of a muscle fiber is inversely related with muscle fiber size (98). A possible explanation for this phenotypical change is that spastic mouse muscles are frequently active due to an overly active stretch reflex or due to tremors.

Here, we show that calf muscles of spastic mice show a fatigue-resistant phenotype. This phenotype resembles that reported for muscles of children with spasticity, for whom higher fatigue resistance has also been shown (99). However, children with spasticity generally have lower scores on the 6-min walking test (100). This discrepancy between the muscle phenotype and endurance gait performance may be explained by impaired efficiency of the gait pattern, limited joint range of motion, reduced balance, and lower motor control in muscles from children with spasticity. Another reason may be that children with spasticity have increased motor unit firing frequencies and increased number of recruited motor units during gait compared with their typical developing peers (101). This may cause fatigue and explain the discrepancy in muscle phenotype and gait performance. Whether effects of recruitment and level of muscle activation of spastic mice during gait are similarly affected in children with spasticity remain to be determined. One explanation may be that changes in muscle phenotype due to oxidative capacity reduced gait performance (i.e., lower gait velocity) in spastic mice. However, studies on muscle fiber type distribution of human spastic muscles are not unequivocal as a result of differences in experimental setup and type of muscles (96, 102–105). Changes in distribution have been suggested to be dependent on the level of functional disability (106). In addition, interventions aiming to reduce spasticity (e.g., botulinum toxin injections) are also known to cause a shift in myosin heavy chain distribution toward more type I muscles fibers (107–109). The above illustrates the impact of severity, age, and treatment on spastic muscle, and highlights that effects of spasticity are co-determined by many other factors, causing substantial variation in the characteristics of spastic human muscle. The present results indicate that spasticity itself without any treatment causes a shift toward a slower phenotype.

### Explanations for the Smaller Muscle Size and Elevated Oxidative Metabolism in Spastic Muscle

The lower mass of spastic soleus and gastrocnemius medialis were in the same order of magnitude as the percentage difference in muscle fiber cross-sectional area. This indicates that spastic muscles fibers had impaired radial growth. Muscle fiber growth is determined by the net rate of synthesis and degradation of contractile and structural proteins. To obtain insight into whether or not protein synthesis was affected by spasticity, we compared the myonuclear density and expression levels of genes involved in protein synthesis and breakdown within the gastrocnemius muscle. The number of myonuclei per muscle fiber was not lower in spastic muscle compared with WT, neither were mRNA expression levels of myostatin and insulin-like growth factor 1 (IGF 1). These results suggest that the capacity for protein synthesis was likely not impaired at the transcriptional level. Regarding the expression of E3 ligases, there were also no differences between spastic and WT muscles. Together, these data suggest that the smaller mass and physiological cross-sectional area of spastic muscles is likely to be as a result of the relatively lower physical activity of the mice and of increased sheltering behavior causing a lower mechanical loading of the muscles. It is well known that mechanical loading stimulates the enzymatic activation of mechanistic target of rapamycin (mTOR), which enhances the rate of translation and protein synthesis (97, 110, 111, 112). The increased mitochondrial content in spastic muscles is likely explained by enhanced transcriptional activity of genes coding for mitochondrial proteins (98). The observation that the number of muscle stem cells was not affected by spasticity indicates that spastic muscles have a typical potential for regeneration in case of injury and also that spastic muscle has sufficient potential for accretion of myonuclei required for longitudinal muscle fiber growth (113).

To our knowledge, muscle gene expression in children has not been extensively studied, neither have mitochondrial nor muscle stem cell content. Gastrocnemius muscle fibers of spastic children show a high variability in cross-sectional area, rather than being smaller (103), particularly in type I muscle fibers. In spastic children, the lack of longitudinal gracilis muscle fiber growth has been suggested to be associated with a lack of addition of sarcomeres in series as well as by reduced myonuclear addition and loss of muscle stem cell numbers (114). Our results suggest that spasticity, whether or not in combination with reduced physical activity, does not alter the muscle stem cell population. However, it is still unknown if, and how, the muscle stem cell population, as well as the myonuclear density, play a role in the ability of muscles to undergo longitudinal growth. The latter might be important, since in the investigated humans, muscles were undergoing lengthening surgery, to treat reduced knee joint range of motion (114).

### Study Limitations

Some limitations of this study should be noted. The uneven male-female ratio in both experimental groups may have increased variability in results of the muscle fiber type distribution, which could have reduced the power to confirm differences between the gastrocnemius medialis of both groups. Nevertheless, the endurance capacity of gastrocnemius medialis muscles was consistently higher in spastic than in WT mice, which supports the shift toward a slower muscle fiber phenotype in spastic mice that became more pronounced with age (Fig. 4).

Moreover, regarding the gene expression data, we determined target mRNA levels relative to those of the housekeeping gene. This approach does not consider any difference in total RNA concentration within spastic and WT muscles. As high oxidative muscle fibers usually have higher RNA concentration (98, 115) and spastic mice appear to have a slower phenotype with smaller muscle fibers, E3 ligase and mitochondrial enzyme activity could be higher in spastic muscle and explain the observed differences in muscle size and metabolism.

### Conclusions

This study showed that glycine receptor subunit-β deficiency in mice results in a series of motor abnormalities, including abnormal gait characterized by toe walking and hopping, impaired hind limb motor function, as well as reduced physical activity. This disturbed motor function is likely the result of spasticity and secondary impairment of skeletal muscle growth. Spastic mice had smaller and weaker plantar flexor muscles, which became more apparent with age. Muscle length of the gastrocnemius medialis muscle was slightly reduced, while passive length-force properties were not affected. The affected force generating capacity is explained by the smaller muscle fiber cross-sectional area, the lower number of sarcomeres in series, and shorter Achilles tendon. Due to this shortening, plantar flexor muscles exert higher forces at lower muscle lengths. In contrast to the effect of maximal force-generating capacity, endurance capacity of spastic muscle was improved, which seems functional and may compensate for the lower force-generating capacity. The glycine receptor subunit-β deficient spastic mouse model captures multiple key features of primary effects of spasticity on muscles function, which may allow for the study of interventions to improve muscle functions, morphological characteristics, and phenotypes in children with spasticity.

### Perspectives and Significance

Clinical interventions aim to improve the mobility and gait of children with spasticity by reducing muscle hyperactivity and increasing the range of joints motion of the affected limbs. However, current interventions are only temporally successful. Moreover, long-term functional limitations are recurrent. Improvement of interventions and development of new drugs require insight into mechanisms underlying spasticity-associated impaired motor function. In vivo studies with typical developing children and children with spasticity are limited with respect to the level of detail of the underlying mechanistic causes. Here, we present a comprehensive characterization of the physical behavior and gait of spastic mice that is related to morphological, histological, and contractile characteristics of ankle plantar flexor muscles of both juvenile and adult spastic mice. Spastic mice have a low muscle force generating capacity due to a smaller physiological cross-sectional area. The muscle length range of force exertion of spastic mice is shifted toward shorter lengths, which is explained by attenuated longitudinal tibia growth. Fatigue resistance of plantar flexor muscles is enhanced, which is explained by a higher mitochondrial content in muscle fibers and relatively higher percentage of slow type muscle fibers. This study shows that in spastic mice, disturbed motor function is the result of hyperactivity of skeletal muscle and concomitant impaired skeletal muscle growth, which progresses with age. We think that our study provides an extensive characterization of the physical performance of the spastic muscle function, which will spur new investigations into medically relevant mechanisms and treatment.

## SUPPLEMENTAL DATA

10.6084/m9.figshare.14838078 Supplemental Table S1: https://doi.org/10.6084/m9.figshare.14838078.

10.6084/m9.figshare.17695577 Supplemental Fig. S1: https://doi.org/10.6084/m9.figshare.17695577.

10.6084/m9.figshare.17695619 Supplemental Fig. S2: https://doi.org/10.6084/m9.figshare.17695619.

10.6084/m9.figshare.17695622 Supplemental Fig. S3: https://doi.org/10.6084/m9.figshare.17695622.

10.6084/m9.figshare.17695625 Supplemental Fig. S4: https://doi.org/10.6084/m9.figshare.17695625.

## GRANTS

Ipsen Innovation funded this study.

## DISCLOSURES

A. Vignaud and M. Kalinichev are employed at Ipsen Innovation. 

## AUTHOR CONTRIBUTIONS

A.V., M.L., M.K., and R.T.J. conceived and designed research; C.R., W.N., and B.K. performed experiments; C.R. and W.N. analyzed data; C.R. and R.T.J. interpreted results of experiments; C.R. prepared figures; C.R., A.V., B.K., M.L., M.K., and R.T.J. drafted manuscript; C.R., A.V., W.N., B.K., M.L., M.K., and R.T.J. edited and revised manuscript; C.R., A.V., W.N., B.K., M.L., M.K., and R.T.J. approved final version of manuscript.
